# Prolotherapy in the Treatment of Knee Osteoarthritis: How to Perform

**DOI:** 10.1002/atn2.70052

**Published:** 2026-05-26

**Authors:** José L. Rocha de Faria, Fabiana Alves C. Menegassi, Sandra T. N. Minamoto, Guilherme Blois V. Pereira, Fábio Henrique P. Videira, Diego G. Severgnine, Caio Gomes de Lima, José Paulo G. Aramburu Filho, Geraldo da R. Motta, João Antônio M. Guimarães, Ronaldo José Faria C. do Amaral, Bianca Gutfilen, Sergio Augusto L. de Souza, Zartur José B. Menegassi

**Affiliations:** ^1^ National Institute of Traumatology and Orthopedics of Brazil Rio de Janeiro Brazil; ^2^ School of Medicine from University of São Paulo USP Ribeirão Preto Ribeirão Preto São Paulo Brazil; ^3^ School of Medicine Federal University Fluminense Niterói Rio de Janeiro Brazil; ^4^ Department of Pathological Anatomy School of Medicine Federal University of Rio de Janeiro Rio de Janeiro Brazil; ^5^ Department of Radiology School of Medicine Federal University of Rio de Janeiro Rio de Janeiro Brazil; ^6^ Department of Orthopedics School of Medicine Federal University of Rio de Janeiro Rio de Janeiro Brazil

## Abstract

The conservative treatment of knee osteoarthritis is a challenge; since it has no cure, many patients do not want or cannot undergo surgical procedures. Among the various options, we have injectable therapies, especially the so‐called “regenerative,” such as the use of hyaluronic acid, orthobiologicals, and prolotherapy. In the latter, its use has increased, either due to ease of application, low cost, or good results, especially with the improvement of pain and function. This technical note describes the protocol for the preparation and injection of intra‐ and extra‐articular prolotherapy in the treatment of knee osteoarthritis. Numerous articles show its benefit both in improving the symptomatic picture and the life quality of affected patients, as long as we follow ideal parameters of the preparation of the substance and how to apply it.

## Graphical abstract

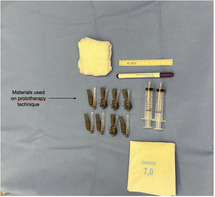

**VIDEO 1:** On this video, we are going to show how to do intra‐ and extra‐articular prolotherapy. The materials used in this procedure are 1 sterile glove, 2 syringes, 1 demographic sterile pen, gauze, 6 25‐gauge needles, alcohol chlorhexidine, and chlorhexidine degerming solution. Here, we can see the patient sitting on the stretcher in a comfortable position, with the knees bent. We start marking the tender points with a demographic pen. While the doctor performs hand asepsis, the assistant does the knee asepsis with chlorhexidine degerming; leave it for 1 min and then remove it with sterile gauze moistened in alcohol chlorhexidine. The doctor puts on the sterile glove and the surgical drape and marks again the tender points with a demographic sterile pen previously located by the patient. We start by the anesthetic button on the skin and subcutaneous tissue at the infiltration site with 2 mL of 2% lidocaine without vasoconstrictor, leave the needle in each tender point and in the intra‐articular point, and prepare the solution (intra‐ and extra‐articular solution). The intra‐articular 25% dextrose is prepared with 5 mL lidocaine 2% and 5 mL of dextrose 50%; the extra‐articular solution is prepared with 7 mL lidocaine 2% and 3 mL of dextrose 50%. Then, we start the infiltration of each point. In this case, we started with the intra‐articular point, we inject 6 mL of the prolotherapy dextrose 25%, always remember to aspirate before injecting the substance, leave the needle to avoid bleeding, engage the syringe of prolotherapy 15% in the first extra‐articular point, inject 2 mL of the substance, disengage the syringe from the needle, and reproduce the same procedure in the other tender points. After infiltrating all the tender points and the intra‐articular point, we start removing each needle and finish the procedure with a simple dressing. Postprocedure, we do 50 cycles of flexion and extension of the patient's knee. Video content can be viewed at https://doi.org/10.1002/atn2.70052.

Intra‐ and extra‐articular injections with platelet‐rich plasma, stem cells, hyaluronic acid, and prolotherapy have been increasingly used for knee ostheoarthritis.^1^, ^2^ Prolotherapy or proliferation treatment is also described as regenerative injection therapy and growth factor stimulation injection therapy. Different classes of proliferative agents exists, including irritants, particulates, osmotics, chemoattractants, and growth factors.^3^, ^4^

Dextrose is one of the most investigated prolotherapy agents, being an osmotic proliferative agent that is safe, inexpensive, and widely available.^5^, ^6^, ^7^, ^8^ Literature shows benefits in pain reduction, stiffness, functions, and quality of life.^9^ Pain in osteoarthritis is related to intra‐ and extra‐articular structures, which justifies treating both.^10^, ^11^, ^12^

Originally developed and applied by George Hackett in 1937,^13^, ^14^ the technique's mechanism is not well known. Proposed mechanisms include reduction of inflammation and stimulation of tissue proliferation, improving repair, pain, and function, especially with the use of injections containing dextrose and lidocaine in painful and/or injured sites.^10^, ^15^, ^16^, ^17^, ^18^

Several studies have shown that dextrose concentrations above 10% have inflammatory activity, which is essential for the production of growth factors necessary tissue repair and regeneration.^10^, ^19^, ^20^, ^21^ Another effect would be the prechondrogenic, with the formation of new cartilage.^22^ Some evidence indicates prolotherapy may be more effective than platelet‐rich plasma, hyaluronic acid, and corticosteroids.^15^, ^16^, ^23^

This technical note describes our methodology for patients aged 40 to 75 years with osteoarthritis diagnosed by the American College of Rheumatology criteria and Kellgren‐Lawrence classification ≤ III, with preserved range of motion. The aim is to standardize intra‐ and extra‐articular techniques, dose, and frequency.

## TECHNICAL NOTE

### Step 1: Preparation of the Solution to Perform Intra‐Articular Prolotherapy and Injection Technique

Prolotherapy is performed with a 25% dextrose solution. It is prepared by mixing 5 mL of 50% dextrose with 5 mL of 2% lidocaine, where 6 mL of this final solution is injected intra‐articular into each affected knee.^10^

**FIGURE 1:**
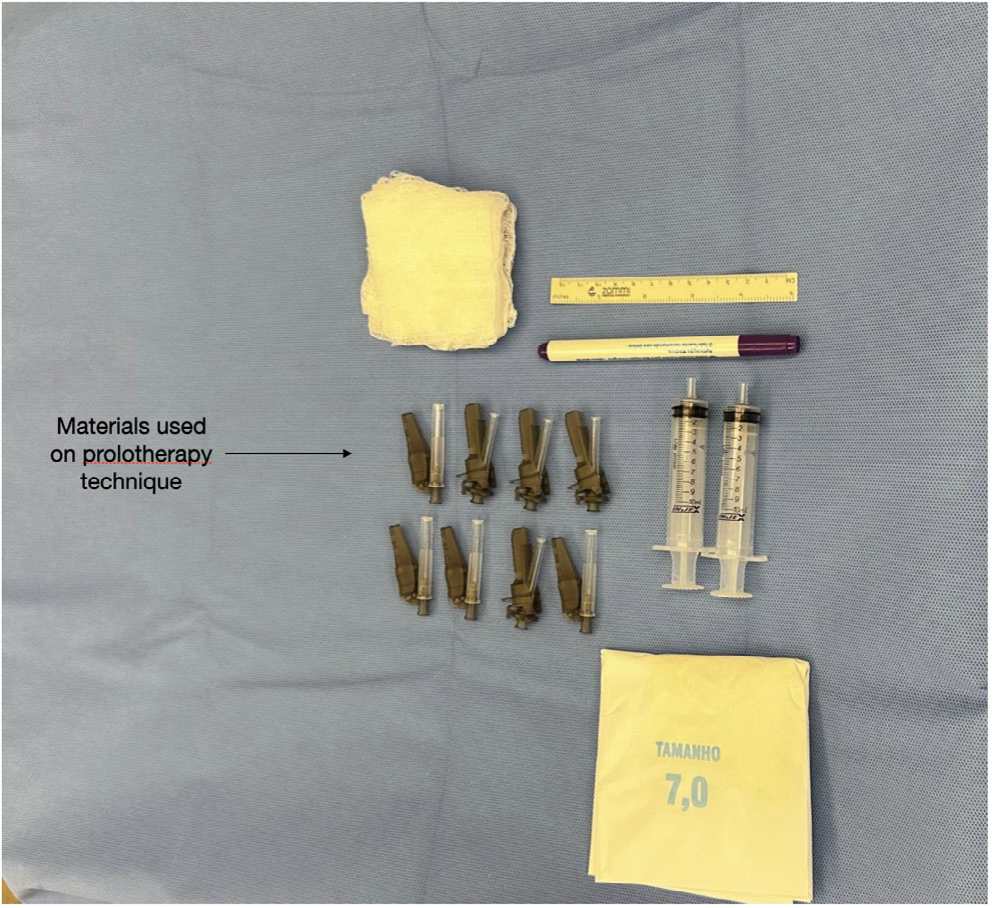
Materials used on intra‐articular and extra‐articular prolotherapy.

**FIGURE 2:**
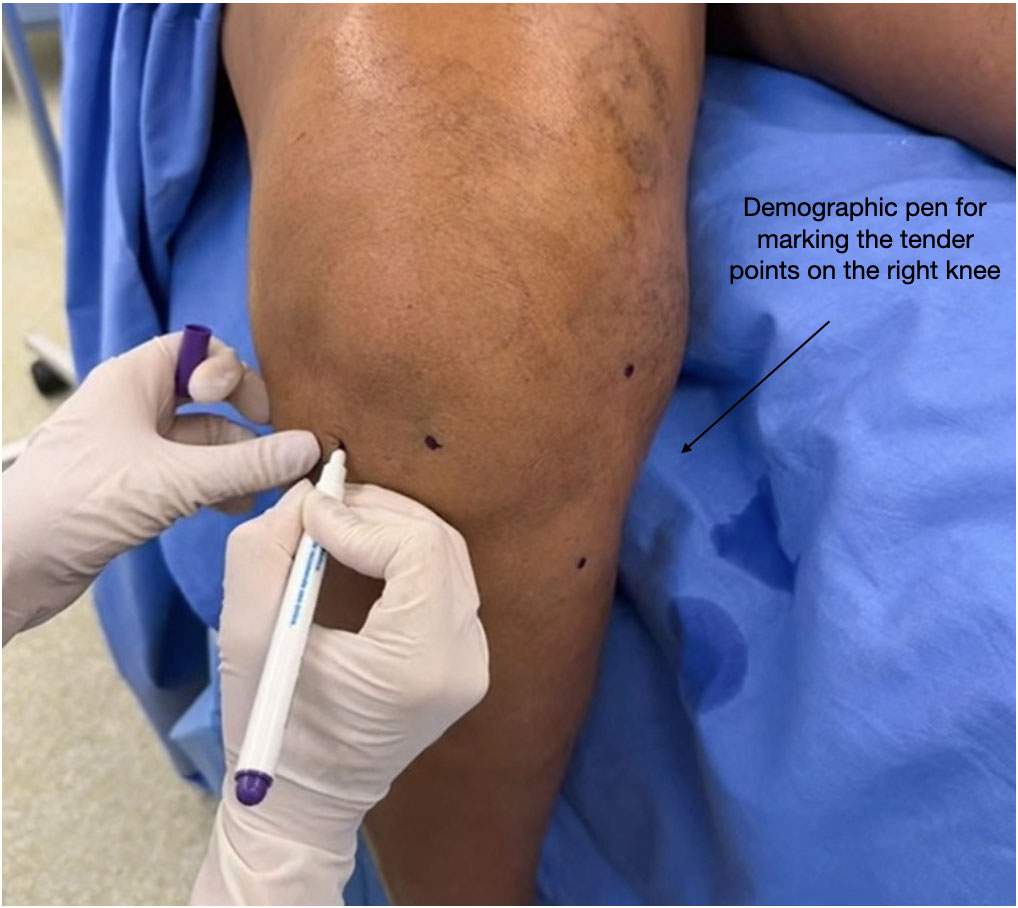
Patient seated in the operating room with the right knee flexed at 90°, the intra‐articular and extra‐articular injection sites were marked using a sterile ballpoint pen for prolotherapy administration—25% intra‐articular and 15% extra‐articular.

**FIGURE 3:**
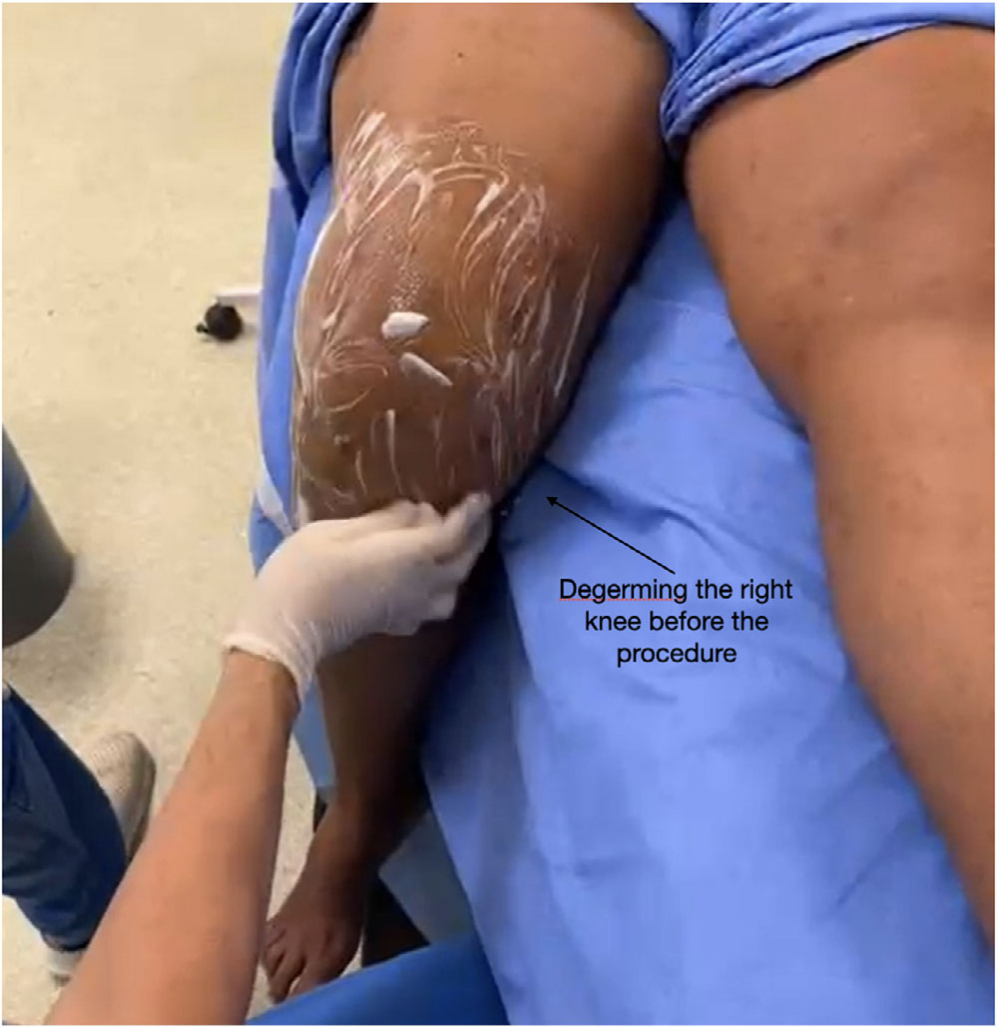
Patient seated in the operating room with the right knee flexed at 90°, and asepsis of the infiltration area was performed with gauze moistened in chlorhexidine degerming.

**FIGURE 4:**
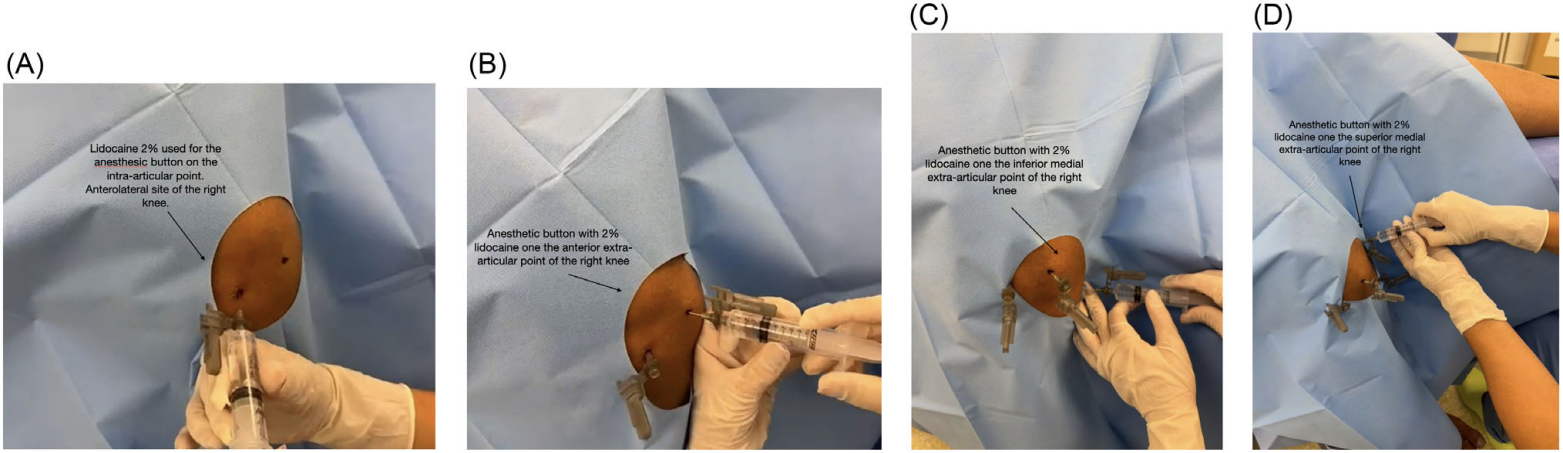
(A) Patient seated in the operating room, right knee flexed at 90°, a sterile fenestrated drape was placed over the knee, and an anesthetic wheal was created with 2 mL of 2% lidocaine without vasoconstrictor at the anterolateral portal of the right knee for intra‐articular prolotherapy. (B) Patient seated in the operating room, right knee flexed at 90°, a sterile fenestrated drape was placed over the knee, and an anesthetic wheal was created with 2 mL of 2% lidocaine without vasoconstrictor on the skin and subcutaneous tissue on the anterior tender point for extra‐articular prolotherapy. (C) Patient seated in the operating room, right knee flexed at 90°, a sterile fenestrated drape was placed over the knee, and an anesthetic wheal was created with 2 mL of 2% lidocaine without vasoconstrictor anesthetic button on the skin and subcutaneous tissue on the medial inferior tender point of the knee for extra‐articular prolotherapy. (D) Patient seated in the operating room, right knee flexed at 90°, a sterile fenestrated drape was placed over the knee, and an anesthetic wheal was created with 2 mL of 2% lidocaine without vasoconstrictor anesthetic button on the skin and subcutaneous tissue on the medial superior tender point of the knee for extra‐articular prolotherapy.

**FIGURE 5:**
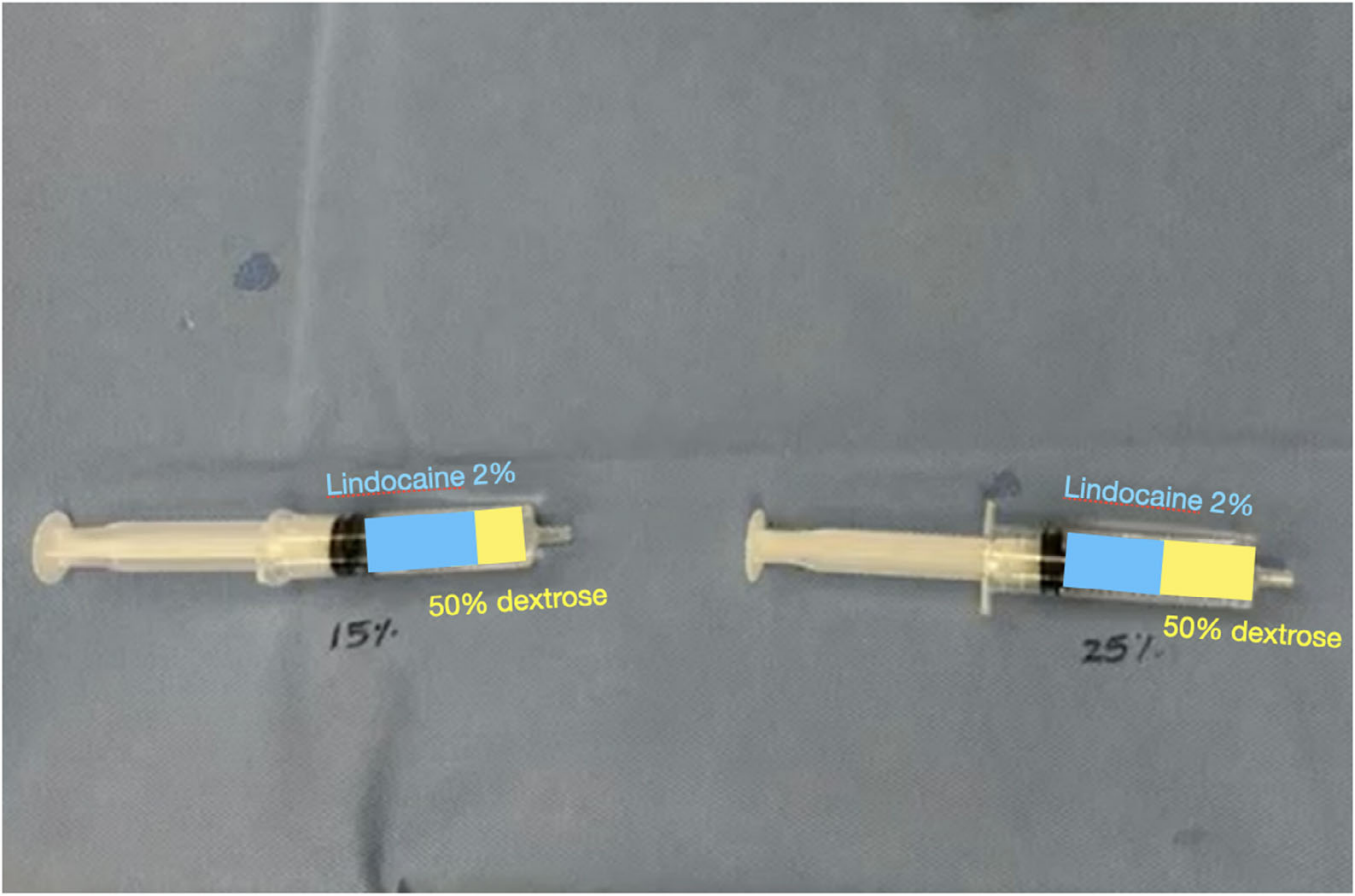
Illustrative image depicting prolotherapy with hypertonic dextrose. The image on the right features a syringe containing the injectate for 25% hypertonic dextrose prolotherapy. The contents are visually represented by 2 colors: blue signifying 2% lidocaine and yellow indicating 50% dextrose. The equal proportions of blue and yellow visually represent the 1:1 volumetric ratio of these 2 agents. Conversely, the image on the left illustrates the injectate for 15% hypertonic dextrose prolotherapy. The contents are again represented by blue (2% lidocaine) and yellow (50% dextrose). In this illustration, the higher concentration of the blue color relative to the yellow visually denotes a proportionally greater volume of lidocaine compared to the dextrose.

**FIGURE 6:**
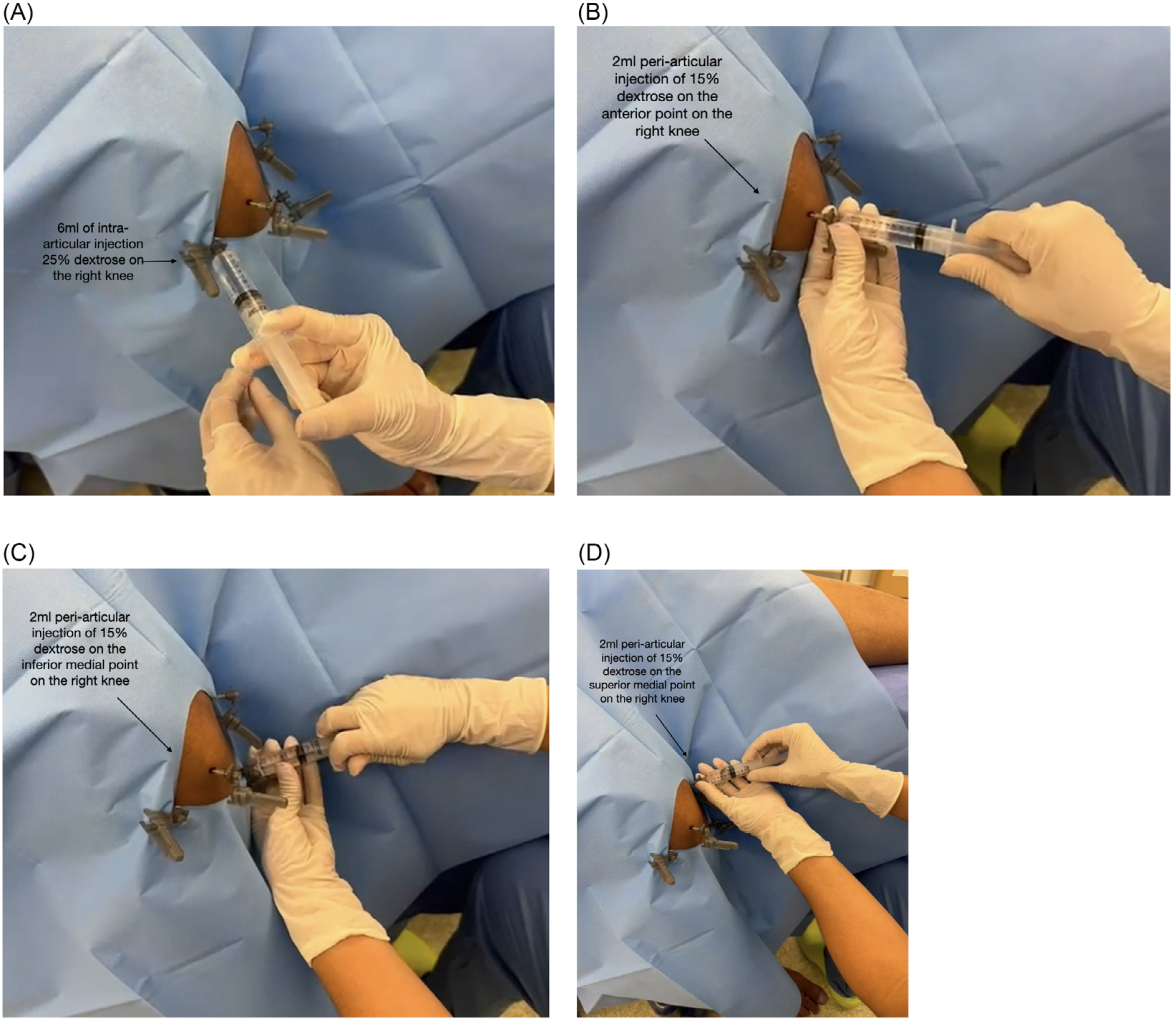
(A) Patient seated in the operating room, right knee flexed at 90°, a sterile fenestrated drape was placed over the knee. Needles are positioned at each tender point, following the prior administration of a local anesthetic wheal using 2% lidocaine without vasoconstrictor. A syringe is positioned at the anterolateral portal of the right knee for the injection of 6 mL of the 25% hypertonic dextrose prolotherapy solution. (B) Patient seated in the operating room, right knee flexed at 90°, a sterile fenestrated drape was placed over the knee. Needles are positioned at each tender point, following the prior administration of a local anesthetic wheal using 2% lidocaine without vasoconstrictor. A syringe is positioned on the anterior point for periarticular injection of 2 mL 15% dextrose. (C) Patient seated in the operating room, right knee flexed at 90°, a sterile fenestrated drape was placed over the knee. Needles are positioned at each tender point, following the prior administration of a local anesthetic wheal using 2% lidocaine without vasoconstrictor. A syringe is positioned on the medial inferior point for periarticular injection of 2 mL 15% dextrose. (D) Patient seated in the operating room, right knee flexed at 90°, a sterile fenestrated drape was placed over the knee. Needles are positioned at each tender point, following the prior administration of a local anesthetic wheal using 2% lidocaine without vasoconstrictor. A syringe is positioned on the medial superior point for periarticular injection of 2 mL 15% dextrose.

**FIGURE 7:**
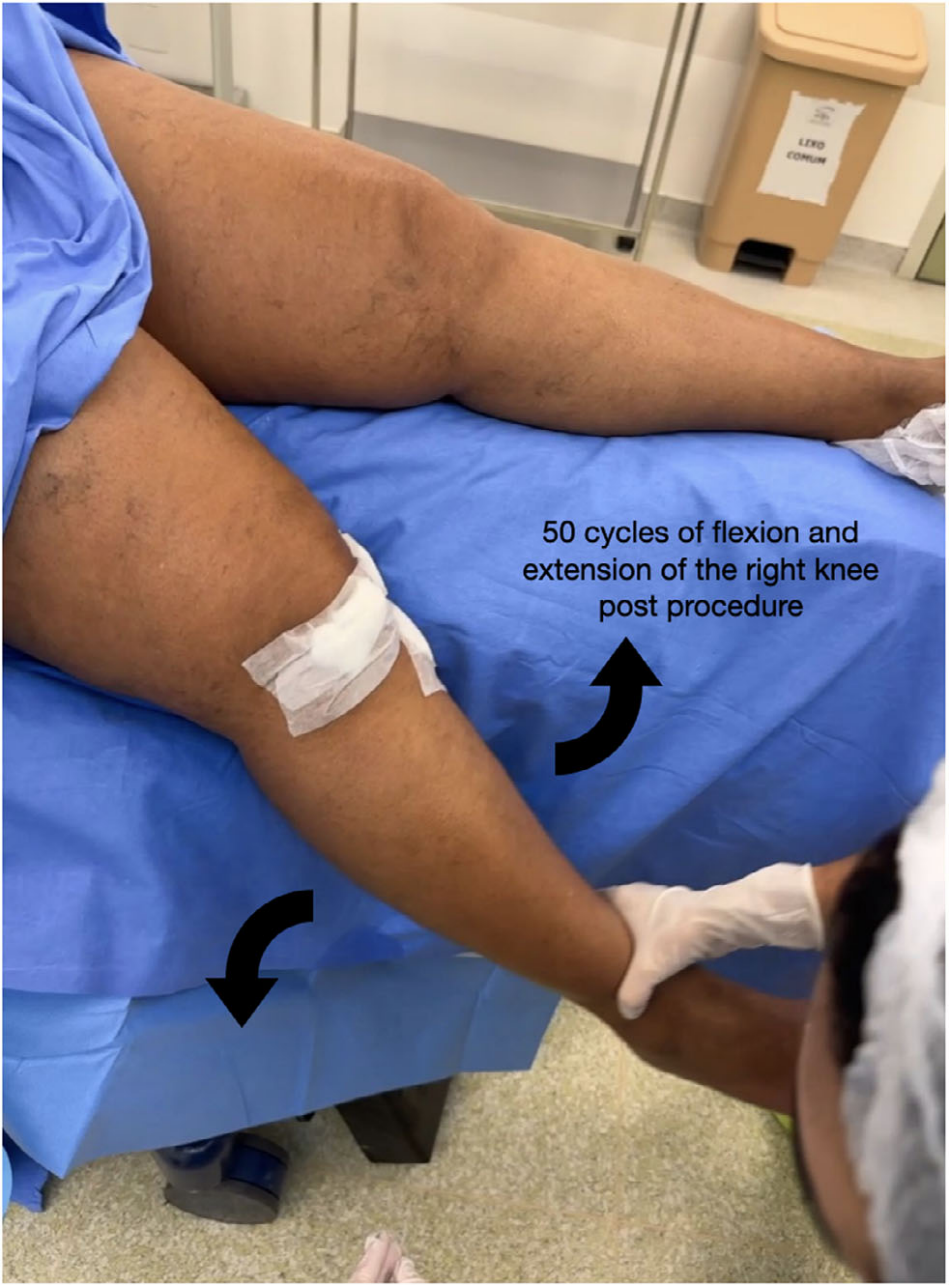
Patient seated in the operating room with a dressing applied over the right knee. The physician performs 50 cycles of knee flexion and extension spread throughout the joint, enhancing its therapeutic effect.

All infiltrations will be performed in the outpatient procedure room and with aseptic technique and all materials for intra‐articular and extra‐articular prolotherapy (Figure 1, Video 1). The technique for the application of intra‐articular prolotherapy is as follows: (1) Position the patient sitting on the stretcher in a comfortable position, with knees bent. (2) Determine the intra‐articular and the tender points for extra‐articular prolotherapy (Figure 2) and clean the area where the application will be carried out with gauze moistened in chlorhexidine degerming (Figure 3). (3) Leave the chlorhexidine on for 1 min and then remove it with sterile gauze moistened with alcohol chlorhexidine. (4) Palpate the knee for unguided infiltration through the anteroinferolateral pathway to the knee with sterile gloves. (5) Perform an anesthetic button on the skin and subcutaneous tissue at the infiltration site with 2 mL of 2% lidocaine without vasoconstrictor (it is aspirated 10 mL of lidocaine 2% in a 10‐mL syringe) (Figure 4A). (6) Leave the needle, prepare the solution (discard 3 mL of lidocaine from the syringe leaving 5 mL; this will be mixed with 5 mL of dextrose 50%) (Figure 5), and attach and inject 6 mL of the substance (Figure 6). (7) Remove the needle. (8) Perform 50 cycles of knee flexion‐extension to distribute the product through the joint (Figure 7). (9) Finish the procedure with a simple dressing. If joint effusion is present, it should be aspirated before infiltration. The patient should be alerted to return to the hospital in case of exacerbated pain and edema or redness in the knee.

### Step 2: Preparation of the Solution to Perform Extra‐Articular Prolotherapy and Injection Technique

Prolotherapy is performed with a 15% dextrose solution. It is prepared by mixing 3 mL of 50% dextrose with 7 mL of 2% lidocaine. This solution is administered extra‐articularly into each affected knee based on the tender points. It was done up to 3 skin punctures using a peppering technique, placing a possible total 6 mL of solution. All infiltrations will be performed in the outpatient procedure room and with aseptic technique. The technique for application extra‐articulate will follow the following steps: (1) Position the patient sitting on the stretcher in a comfortable position, with knees bent. (2) Mark the tender points with a demographic pen (Figure 2). (3) Clean the area where the application will be carried out with gauze moistened in chlorhexidine degerming (Figure 3). (4) Leave the chlorhexidine on for 1 min and then remove it with sterile gauze moistened with alcohol chlorhexidine. (5) Mark again the tender points located previously by the patient with a demographic sterile pen. (6) Put the surgical drape. (7) Perform an anesthetic button on the skin and subcutaneous tissue at the infiltration site with 2 mL of 2% lidocaine without vasoconstrictor (Figure 4B‐D). (8) Leave the needle, prepare the solution (7 mL of lidocaine 2% will be mixed with 3 mL of dextrose 50%) (Figure 5), and attach and inject 2 mL of the substance up to 3 subdermal injections (2.0 mL of 15% solution per point) (Figure 6B‐D). (9) Remove the needle. (10) Finish the procedure with a simple dressing. The patient should be alerted to return to the hospital in case of exacerbated pain and heat or redness in the knee.

Steps 1 and 2 are done subsequently.

Post procedure recommendations fifty cycles of flexion‐extension of the knee 3 times a day for 3 days and physical activity should not be performed for 5 days. In case of pain, dipyrone and ice 3 times a day for 20 min.

## DISCUSSION

Prolotherapy with hypertonic dextrose has shown symptom improvement in patients with knee osteoarthritis. Technique standardization on text, pictures, and videos helps other researchers around the world to replicate this important therapy. The simplicity of the preparation and the low cost are relevant factors that contribute to disseminate this technique.

In a recent literature review study, one of the difficulties is the different dose protocols of dextrose used; 4 trials were conducted using 5 mL of dextrose, whereas 1 trial used 10 mL, another used 3.5 mL, 1 used 2 mL, 1 used 6 mL, and 2 used 7 mL.^9^ But the most important factor for efficacy of this treatment seems to be the dextrose concentrations above 10% that have inflammatory activity, which is essential for the production of growth factors necessary for the process of tissue repair and regeneration what we have accomplished in this technical note.^10^, ^19^, ^20^, ^21^

The use of intra‐articular anesthetic, especially in arthroscopic procedures with local anesthesia, as well as in postoperative pain management, is very common in clinical practice.^24^ Chondrolysis has been reported in an increased number of patients where continuous infusion of anesthetic has been used especially when they involved the glenohumeral joint, which was considered more vulnerable to this effect (about 90% of cases).^25^ Despite this, cases of chondrolysis have also been reported on the knee.^26^ A direct correlation of anesthetic concentration and frequency of side effects has been shown.^27^

It is known that lidocaine is the anesthetic with toxic effects.^28^, ^29^ However, according to Vrachnis et al.,^30^ in 2024, a single intra‐articular dose of lidocaine may not cause any toxic effect on the cartilage in the short term. McCutchan et al.,^31^ in 2019, reported the absence of cytotoxic and inflammatory effects following in vitro exposure of chondrogenically differentiated human mesenchymal stem cells to adenosine, lidocaine, and Mg^2+^ solution and did not stimulate secretion of key inflammatory mediators or matrix‐degrading matrix metalloproteinases that are believed to delay healing and promote secondary injury.

The risks of this technique are local pain and slight bleeding at the sites where the needle is introduced with full resolution up to 72 h after the procedure,^32^ knee stiffness, and infection at the injection site, as with any injection, although the risk is low when a sterile technique is used. The main advantages of this technique are its easy reproducibility, low cost, and good efficacy in improving pain and function, contributing to the conservative management of knee osteoarthritis. It can be performed with simple, inexpensive materials, making it feasible for a much broader patient population due to its minimal cost. As the literature has shown, the association of intra‐articular and extra‐articular prolotherapy has more effective results in the osteoarthritis pain.^10^, ^11^, ^12^ Although, as with any procedure, there are advantages and disadvantages (Table 1), this treatment is considered safe. Some practical tips can be applied to assist in clinical practice (Table 2).

**TABLE 1:** Pearls and Pitfalls of Prolotherapy on the Treatment of Knee Osteoarthritis

Pearls	Pitfalls
A prior anesthetic wheal should be performed, as the procedure can be painful without it and may be poorly tolerated by patients	Always aspirate before injecting to avoid inadvertent intravascular administration of the anesthetic or proliferant solution
Flexion‐extension exercises performed immediately after the procedure and during the first 3 postoperative days help the proliferant solution to spread throughout the joint, enhancing its therapeutic effect	If moderate to high resistance is encountered on the syringe plunger during intra‐articular injection, reposition the needle, as intra‐articular infiltration should offer minimal resistance
Keeping the needles in place immediately after anesthetic infiltration minimizes tissue trauma and reduces patient discomfort	

**TABLE 2:** Advantages and Disadvantages of Prolotherapy on the Treatment of Knee Osteoarthritis

Advantages	Disadvantages
Low cost	Lack of standardization in the treatment protocol
Easy reproducibility	Pain and discomfort
Improvement of pain and knee function, leading to enhanced quality of life	Bleeding

In conclusion, we observed that prolotherapy is a procedure with a fast‐learning curve, with little morbidity for patients, low cost, and, after correct indication, preparation, and application, a good effectiveness in symptomatic improvement of patients with knee osteoarthritis, which is in line with numerous authors.^33^, ^34^, ^35^, ^36^

This technical note sought to contribute to the standardization of the use of prolotherapy in knee osteoarthritis, with a future result of presenting its results in a larger series.

## DISCLOSURES

The author (J.L.R.F.) declares the following financial interests/personal relationships which may be considered as potential competing interests: J.L.R.F. reports a relationship with Sintegra Surgical Sciences that includes consulting or advisory. The other authors (F.A.C.M., S.T.N.M., G.B.V.P., F.H.P.V., D.G.S., C.G.L., J.P.G.A.F., G.R.M., J.A.M.G., R.J.F.C.A., B.G., S.A.L.S., Z.J.B.M.) declare that they have no known competing financial interests or personal relationships that could have appeared to influence the work reported in this article.
